# Pipelines for lymphocyte homeostasis maintenance during cancer immunotherapy

**DOI:** 10.3389/fimmu.2025.1522417

**Published:** 2025-03-24

**Authors:** Bensu Du, Jin Geng, Bin Wu, Houru Wang, Ru Luo, Hanmeng Liu, Rui Zhang, Fengping Shan, Lei Liu, Shuling Zhang

**Affiliations:** ^1^ China Medical University, Shenyang, China; ^2^ Department of Ophthalmology, The First Hospital of China Medical University, Shenyang, China; ^3^ Department of Hematology, Shengjing Hospital of China Medical University, Shenyang, China; ^4^ Northeast Yucai Foreign Language School, Shenyang, China; ^5^ Jinzhou Medical University, Jinzhou, China; ^6^ Department of Immunology, School of Basic Medical Science, China Medical University, Shenyang, China; ^7^ Department of General Surgery, Shengjing Hospital of China Medical University, Shenyang, China; ^8^ Department of Oncology, Shengjing Hospital of China Medical University, Shenyang, China

**Keywords:** CD8 + T cells, tumor-associated high endothelial venules, immunotherapy, tumor-associated lymphatic vessels, lymphocytes

## Abstract

In general, increasing lymphocyte entry into tumor microenvironment (TME) and limiting their efflux will have a positive effect on the efficacy of immunotherapy. Current studies suggest maintenance lymphocyte homeostasis during cancer immunotherapy through the two pipelines tumor-associated high endothelial venules and lymphatic vessels. Tumor-associated high endothelial venules (TA-HEVs) play a key role in cancer immunotherapy through facilitating lymphocyte trafficking to the tumor. While tumor-associated lymphatic vessels, in contrast, may promote the egress of lymphocytes and restrict their function. Therefore, the two traffic control points might be potential to maintain lymphocyte homeostasis in cancer during immunotherapy. Herein, we highlight the unexpected roles of lymphocyte circulation regulated by the two gateways for through reviewing the biological characters and functions of TA-HEVs and tumor-associated lymphatic vessels in the entry, positioning and exit of lymphocyte cells in TME during anti-tumor immunity.

## Introduction

The recruitment of lymphocytes into the tumor microenvironment (TME) is an essential way to enhance the efficacy of the treatments involving immune checkpoint blockade (ICB), vaccines, or adoptive T cell immunotherapy (1–3). Before infiltrating into TME, the lymphocytes usually migrate to the blood vessels and then extravasate from them (4). High endothelial venules (HEVs) are specialized vessels dedicated to lymphocyte recruitment in lymph nodes and other lymphoid organs (5, 6). Lymphocytes including CD8^+^ T cells can enter tumor tissue through HEVs and then participate in antitumor activities. Previous studies revealed that MECA-79^+^ tumor-associated HEVs (TA-HEVs) were present in some types of human solid tumors, and the density of TA-HEVs in TME was associated with the numbers of CD3^+^ CD8^+^ T cells and CD20 B cells (4, 7, 8). Subsequently, another study evidenced that the cycling lymphocytes in blood could enter the tumor tissue continuously through TA-HEVs, thus enhancing the immune response during antitumor activities (9).

**Figure 1:**
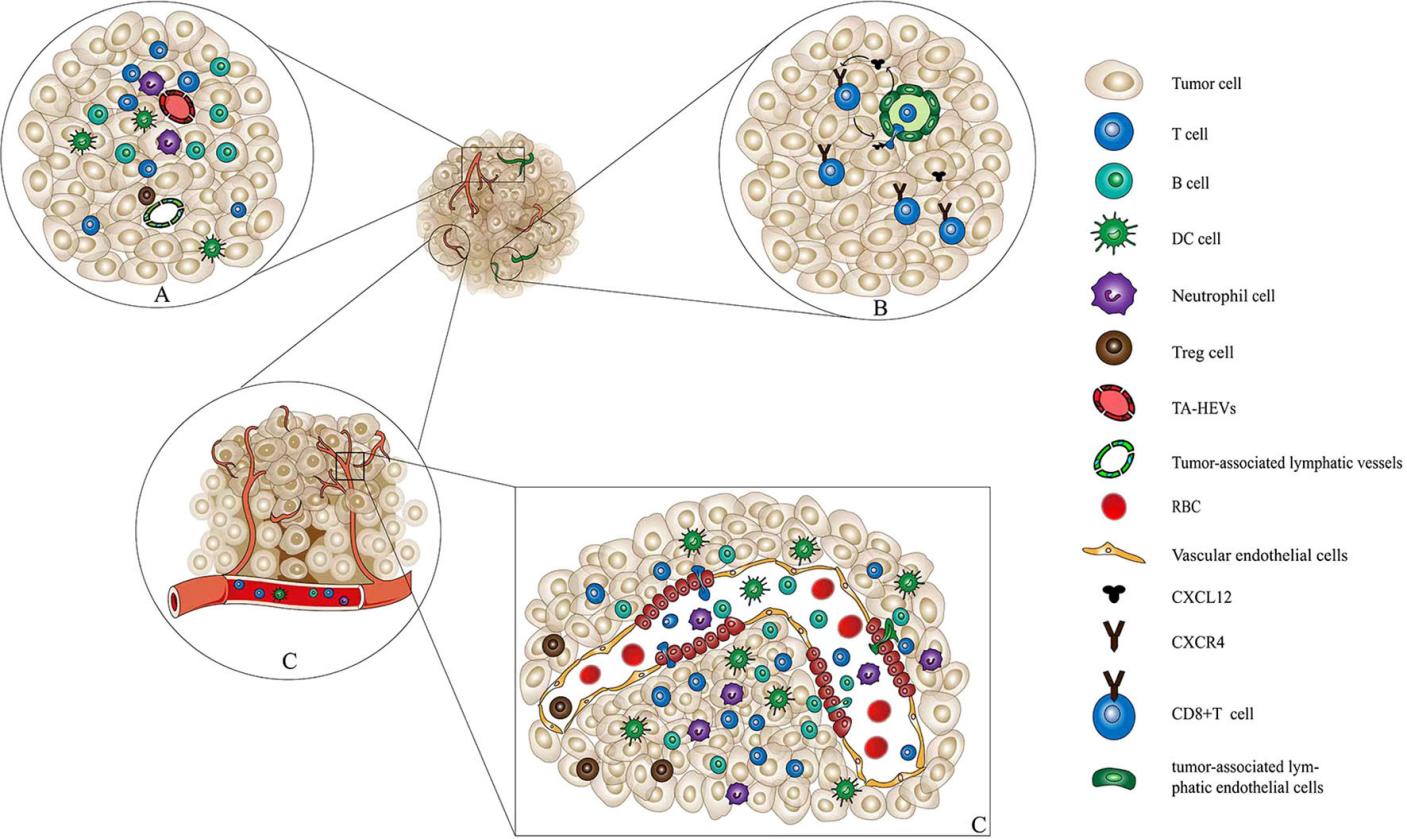
Interaction among immune cells, TA-HEVs, and tumor-associated lymphatic vessels. **(A)** Interaction among immune cells, TA-HEVs, and tumor-associated lymphatic vessels. In tumor tissue, immune cells including CD8^+^ T cells, CD4^+^ T cells, and DCs infiltrate tumor tissue through tumor-associated high endothelial venules and exit through tumor-associated lymphatic vessels. In this way, the immune cells maintain their number and function of infiltration in tumor tissue. **(B)** Tumor-associated lymphatic vessels mediate the exit of CD8^+^ T cells from the tumor tissue. Tumor-associated lymphatic endothelial cells can secrete the cytokine CXCL12. Further, CXCL12 binds to the receptor CXCR4 on the surface of CD8^+^ T cells, inducing CD8^+^ T cells to approach and pass through the lymphatic endothelial cell space, and finally exit the tumor tissue. **(C)** HECs and the structure of vascular endothelial cells and immune cells through the HECs into tumor tissue. HECs are transformed by capillary endothelial cell migration. Immune cells such as lymphocytes, mediated by chemokines, undergo processes such as receptor recognition, adhesion, and rolling to infiltrate tumor tissues through paracellular pathways. TA-HEVs, tumor-associated high endothelial venules; DCs, dendric cells; CXCL12, C-X-C motif chemokine ligand 12; CXCR4, C-X-C motif chemokine receptor 4; HECs, high endothelial cells.

The accumulation of tumor-specific CD8^+^ T cells within TME is essential for the efficacy of immunotherapy. Besides increasing the infiltration of T cells into TME, reducing the leakage of effecter T cells from TME is another way to enhance the effects of antitumor activities. Studies exploring the exit of lymphocytes from tumor tissue mainly focused on the lymphatic vessels regulated by antigen contact (10). The C-X-C motif chemokine ligand 12(CXCL12) is produced by tumor-associated lymphatic endothelial cells. The C-X-C motif chemokine receptor 4 (CXCR4) on the surface of T cells binds to and recognizes CXCL12 and then leaves the tumor along the lymphatic vessels and is sealed around the tumor (**Figures 1A, B**).

Therefore, strategies limiting the leakage of effector T cells from TME while increasing the infiltration of T cells into TME can help improve the efficacy of immunotherapy. We conducted this systematic review on both the entry and exit of lymphocytes from TME, aiming to explore the potential avenues to enhance the efficacy of immunotherapy.

## TA-HEVs: a pipeline for the entry of T cells into TME

### Structure of HEVs

As specialized postcapillary venules, HEVs are primarily located in other secondary lymphoid tissues except the spleen (11). High endothelial cells (HECs) are unique microstructures in HEVs, characterized by highly columnar and approximately cuboidal endothelial cells, with a width of 7–10 μm and a height of 5–7 μm (12–14), thus distinguishing them from other common endothelial cells. HECs occupy most of the luminal space, and hence the lumen of HEVs is narrow or even closed and covered with a thick basement membrane and a perivascular sheath (5, 15, 16). HECs have a large round nucleus with one or two nucleoli and abundant organelles such as mitochondria, rough endoplasmic reticulum, ribosomes, and Golgi complex. They show the characteristics of secretory cells with highly fast metabolism (15, 17–19). Ultrastructural studies revealed that HECs have a thick carbohydrate-rich glycocalyx coating on their luminal surface (20–22). In addition, sulfated carbohydrates and glycoproteins are important recognition determinants of L-selectin in lymphocytes (23). Moreover, the glycocalyx in HECs may also facilitate the retention of secreted molecules on the luminal surface of HECs (14, 20).

As HECs are specialized capillary endothelial cells, we focused on their mode of transformation. The pathway represented by lymphotoxin and tumor necrosis factor α (TNF-α), which are mainly secreted by activated lymphocytes and natural killer cells (NK cells), is the most critical signaling pathway during HEVs formation (4). Lymphotoxin α3(LTα3) or TNF-α binds to tumor necrosis factor receptor 1 or 2 (TNFR1/2), whereas LTα1β2 or tumor necrosis factor superfamily 14 (LIGHT) signals bind to LTβR. TNF-α or LTα3 drives spontaneous HEVs formation, whereas LTα1β2 and LIGHT are the major inducers of HEVs. After HEVs formation, their special morphology also needs to be maintained (24, 25). LTβR signaling is important in maintaining the HEVs phenotype because LTαβ/LTβR signaling can maintain the cube-like morphology of HECs (26, 27). Sphingosine-1-phosphate (S1P)–S1PR1 axis also plays a role in regulating HEVs maintenance (28–30). The complex relationship between LTβR signaling and TA-HEVs plays an important role in the differentiation and growth of TA-HEVs (27, 31). As such, LTβR agonists are potent inducers of TA-HEVs in tumors (32).

### Function of HEVs and mechanisms of lymphatic homing

The special structure of HECs contributes to the special role of HEVs in lymphocyte homing and recycling (33–35). Lymphocytes may enter lymph nodes via two transendothelial migration pathways: the paracellular route (through intercellular spaces) and the transcellular route (through endothelial cells). However, the paracellular route is predominant (36–39). First, the ligands of L-selectin synthesized by HECs are processed by the Golgi complex, stored in secretory granules, and released on the luminal surface of HECs for recognition by homing receptors on the surface of passing lymphocytes to initiate the homing of lymphocytes (40). Next, the circulating lymphocytes in the blood bind to the 6-sulfo sialyl Lewis X motif modifying HEVs via L-selectin, and then tether and roll on the HEVs wall, allowing them to immobilize with heparan sulfate and interact with chemokines on the luminal surface of HEVs (41, 42). These chemokines can mediate the activation of integrins essential for lymphocyte arrest in HEVs (43–47). Integrin lymphocyte function–associated antigen 1 (LFA1) is the major integrin responsible for T and B cell arrest in HEVs in peripheral lymph nodes. The combination of the shear stress of blood flow and the G protein-coupled chemokine receptor signaling induces a conformational change in the LFA1 molecule, leading to the firm adhesion of lymphocytes to intercellular adhesion molecules 1 and 2 (ICAM1 and ICAM2) expressed on HECs (44, 46, 48). After stable arrest, the lymphocytes crawl along the luminal surface of the HEVs in search of suitable transport sites. Thereafter, they migrate through transcellular or paracellular pathways (49, 50). During migration through the paracellular pathway, the high columnar HECs rapidly close the opened intercellular space on the side of the lumen after lymphocyte passage, thus minimizing the leakage of intravascular fluid (36, 51).

### Distribution and role of TA-HEVs

The antitumor immune response primarily depends on the activity of tumor-specific lymphocytes that can recognize and eliminate tumor cells. TA-HEVs are a major gateway for lymphocyte infiltration into human tumors. They are usually found in areas with B cell–rich tertiary lymphoid structures (TLS) as well as in areas with high densities of T cells and mature dendritic cells (DCs) (52, 53). TA-HEVs can be induced in different types of tumors (54, 55). However, MECA-79^+^ TA-HEVs are sometimes formed spontaneously in the TME even without any treatment (55–57). MECA-79^+^ TA-HEVs facilitate the recruitment of naive lymphocytes to tumors (4, 58). Further studies suggested that a high density of MECA-79^+^ TA-HEVs was associated with increased infiltration of naive and central memory T cells into TME (4, 7, 52). Therefore, an increase in the number of T cells in different morphological stages within the tumor is proposed to accelerate and foster antitumor response (59, 60). The induction of MECA-79^+^ TA-HEVs is also observed in a number of mouse tumor models. This is associated with the infiltration of T cells, especially CD8^+^ T cells, and the inhibition of tumor growth (61, 62). Moreover, increasing the differentiation and maturation of HEVs in tumors can reduce CD8^+^ T cell depletion and lead to an increase in the proportion of stem-like CD8^+^ T cells (52) (**Figure 1C**).

### Relationship between immunotherapy and TA-HEVs

**Figure 2:**
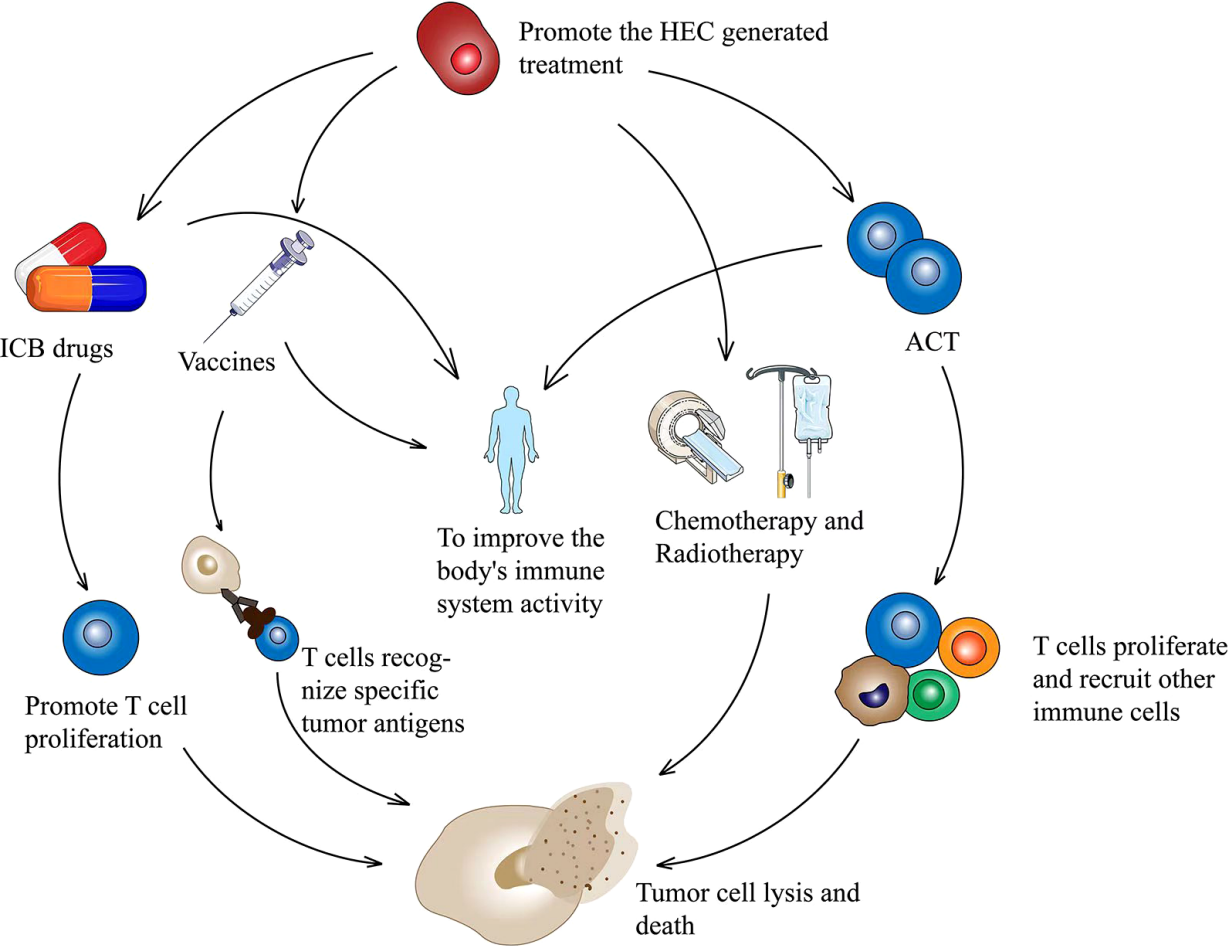
HECs production therapy in combination with other treatment options to inhibit tumor cell proliferation. Therapies combined with ICB promote T cell activation and proliferation. A combination with tumor vaccine promotes T cell specificity and kills tumor cells. HECs therapy combined with ACT promotes the proliferation of tumor killer T cells and recruits other immune cells. A combination of chemotherapy and radiotherapy effectively kills tumors. HECs, high endothelial cells; ICB, immune checkpoint blockade; ACT, adoptive T cell therapy.

**Figure 3:**
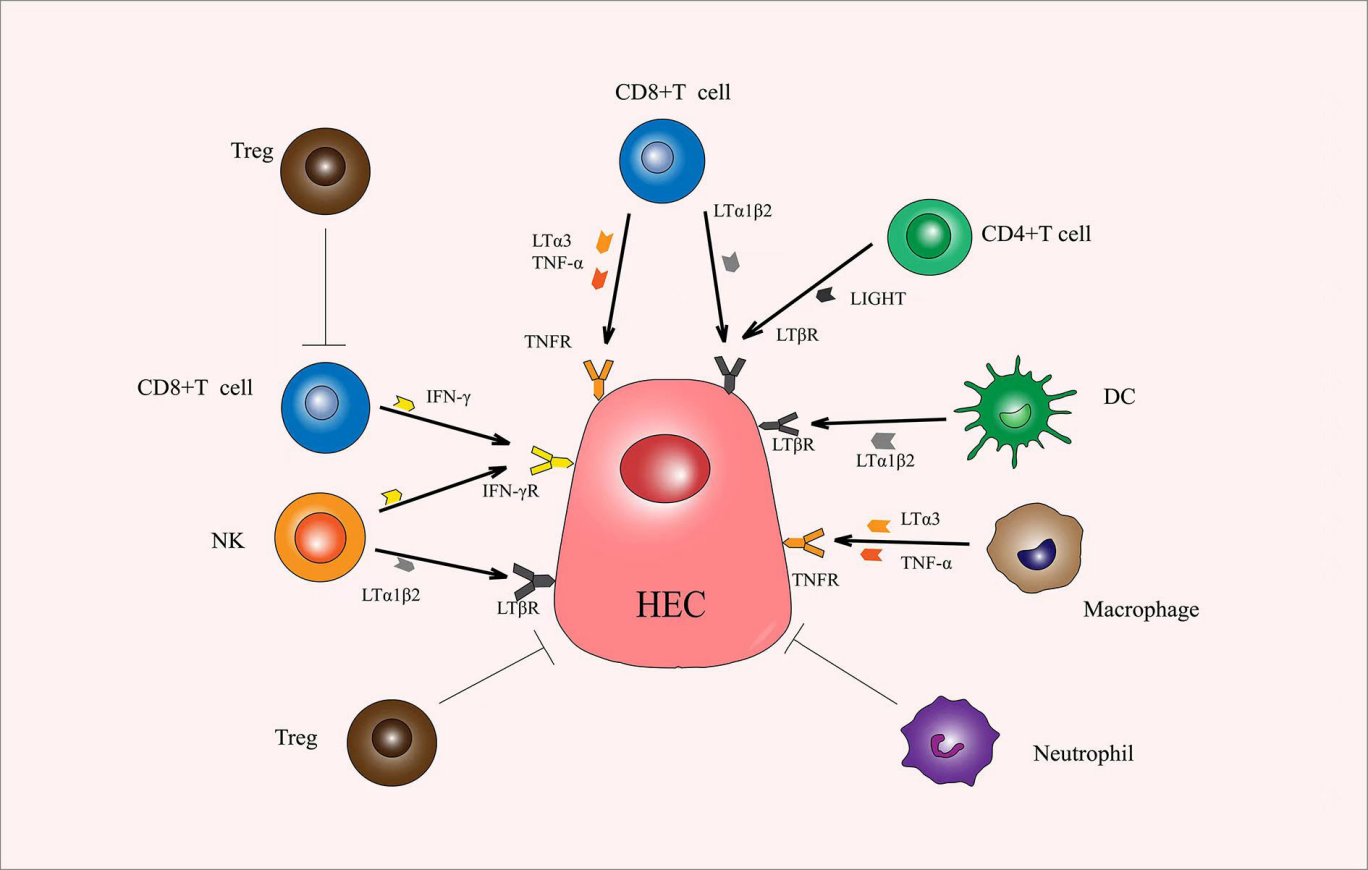
Immune cells produce various cytokines that promote or inhibit the production of HECs. CD8^+^ T cells play an important role in the production of HECs. LTα1β2, LTα3, TNF-α, and IFN-γ secreted by CD8^+^ T cells promotes the production and differentiation of HECs. CD4^+^ T cells secrete LIGHT, DCs secrete LTα1β2, macrophages secrete LTα3 and TNF-α, and NK secrete LTα1β2 and IFN-γ, and all can promote the production and differentiation of HECs. Tregs and neutrophils can exert a direct inhibitory effect. Also, Tregs can inhibit the function of CD8^+^ T cells. HECs, high endothelial cells; LTα1β2, lymphotoxin α1β2; LTα3, lymphotoxin α3; TNF-α, tumor necrosis factor α; IFN-γ, interferon γ; LIGHT, tumor necrosis factor superfamily 14; DCs, dendric cells.

Theoretically, the efficacy of immunotherapy can be potentiated by the increased generation of HEVs, which in turn promotes lymphocyte entry into TME. In fact, many studies have suggested that the therapeutic induction of TA-HEVs in tumors might enhance the trafficking of endogenous lymphocytes, as well as adoptive transferred lymphocytes, and improve the efficacy of various cancer therapies, including immunotherapy with immune checkpoint inhibitors (ICIs), adoptive T cell therapy (ACT), vaccines, and potential targeted and conventional cancer therapies (radiotherapy and chemotherapy) (2, 63) (**Figure 2**). Additionally, the immune cells, especially lymphocytes, may also have an effect on TA-HEVs. CD8^+^ T cells may be the major inducers of TA-HEVs in tumors (64, 65). CD8^+^ T cells can produce LTα3, LTα1β2, TNF-α, IFN-γ, and other signals that induce the activation of lymphotoxin beta receptor (LTβR), thus contributing to the production of TA-HEVs (66). NK cells express LTα1β2 and IFN-γ. Follicular helper T cells express LIGHT. Macrophages express TNF-α, and LTα3 plays a role in contributing to the production of TA-HEVs. In other tumor models, the generation of TA-HEVs also requires the participation of B cells and DCs (65, 67–69). Some DCs can express LTβR and directly promote the differentiation of DC-dependent HEVs (31). CD11c integrin is important in DC physiology; Moussion and Girard demonstrated in mice that CD11c cells were essential for HEVs formation (6, 70). Thus, a larger number of CD11c cells could trigger increased HEVs formation (70). The additional regulatory role of DCs is to promote the growth of TA-HEVs in a vascular endothelial growth factor (VEGF)-dependent manner (71, 72). Regulatory T cells (Tregs) appear to limit the development of TA-HEVs in tumors, but their mechanism of action remains unclear (69, 73). The appropriate ways to treat LTβR should be explored, and the number of CD8^+^ T cells, DCs, and Tregs should be reasonably regulated (**Figure 3**). Tumor progression may also affect the presence of TA-HEVs, which needs further investigation. Since the last decades, ICB therapy, including programmed death 1 and programmed death-ligand 1(PD-1 and PD-L1) blockade treatments, has exhibited positive effects in antitumor immunotherapy for several solid tumors (2). Therefore, we focused on the association between ICB therapy and TA-HEVs. Previous studies suggested that anti-PD-1 therapy might have the potential to promote tumor immunity by stimulating the formation of TA-HEVs (74). At the same time, the formation of TA-HEVs can cooperate with anti-PD-1 therapy to promote the infiltration of lymphocytes, so that they can play a potent immune role against tumors (5, 75). Some other ICIs, such as anti-CTLA-4, can increase the abundance of TA-HECs and tumor-infiltrating CD4^+^ and CD8^+^ T cells (52, 74). A combination of antiangiogenic therapy and ICB also achieved similar efficacy, thus improving the infiltration of CD8^+^ T cells from the periphery (52). Some mouse experiments revealed a significant increase in the homing efficiency of TA-HEV-mediated lymphocytes after ICB treatment, possibly contributing to an increase in the tumor-specific T cell repertoire (76, 77).

## Tumor-associated lymphatic vessels: the pipeline for the exit of T cells from TME

### Role of Tumor-associated lymphatic vessels

Tumor-associated lymphatic vessels carry interstitial fluid and leukocytes unidirectionally from peripheral tissues to lymph nodes and are mainly involved in the generation, maintenance, and regression of adaptive immunity (78). Tumor-associated lymphatic vessels and their transport function are important conditions for controlling the exit of lymphocytes from tumor tissues. Tumor-associated lymphatic vessels can exert immunosuppressive effects, cross-present antigens, and limit CD8^+^ T cell-dependent tumor control in an interferon-γ (IFN-γ)-dependent manner, directly leading to tumor immune escape (79–83). However, the lymphatic system also contributes to regulating the diversity and functional status of CD8^+^ T cells within the tumor (84).Therefore, the inhibition of CD8^+^ T cell shedding by the lymphatic system is an important control point to enhance the response to immunotherapy.

### Mechanisms of T cells exiting the tumor through tumor-associated lymphatic vessels

The exit of lymphocytes from tumors through tumor-associated lymphatic vessels is mainly regulated by various chemokines. For example, chemokine12 (CXCL12) and its receptor CXCR4 mediated the exit of CD8^+^ T cells from the tumor through tumor-associated lymphatic vessels. In this process, CXCL12, a ligand for CXCR4, has been shown to promote the egress of DCs and B-lymphocytes (85, 86). CXCL12 can also promote the exit of CXCR4 CD8^+^ T cells from the tumor, and lymphangiogenic CXCL12 is sufficient to affect the accumulation and location of T cells in the tumor (10). Antigen contacts regulate surface CXCR4, and therefore CD8^+^ T cells are sensitive to CXCL12. The peritumoral tumor-associated lymphatic vessels direct the location and retention of CD8^+^ T cells in the TME by expressing CXCL12, which recruits and ultimately egresses a wide range of functional tumor-specific CXCR4 CD8^+^ T cells (87). Blocking CD8^+^ T cell shedding through this pathway can improve local tumor control and ICB response (88, 89). Atypical chemokine receptor 3 (ACKR3) is a decoy receptor for CXCL12. CD8^+^ T cells can modulate their CXCL12 sensitivity *in vivo* by downregulating the expression of surface CXCR4 and upregulating the expression of ACKR3 [ (10, 90). Therefore, the retention of memory and effector CD8^+^ T cells in tumor tissue can be promoted by downregulating the CXCR4 expression or reducing its sensitivity to CXCL12, thus allowing it to play an immune role against the tumor for a longer time (87).

### Co-regulation of CXCR4 with intratumoral T cell differentiation to determine the repertoire of retained lymphocytes

Not all the T cells in the tumor are exported under the action of CXCL12–CXCR4, but selectively. Besides the role of CXCR4, T cell differentiation is also coordinated to participate in exported and retained lymphocyte screening. Exported effector CD8^+^ T cells have various transcriptional modules with retained effector CD8^+^ T cells (91). Tumor-retained effector CD8^+^ T cells are enriched for transcriptional modules related to hypoxia, leukocyte activation, and extracellular matrix organization, whereas exported CD8^+^ T cells are enriched for transcriptional modules related to leukocyte migration, glutamate receptor signaling, and branched-chain amino acid catabolism. The retained intratumoral CD8^+^ T cells are enriched for a gene set that is upregulated upon exhaustion (e.g., PD-1, nuclear receptor subfamily 4,group a(Nr4a2), cytotoxic T-lymphocyte-associated protein 4(Ctla4), and T cell immune receptor with Ig and ITIM domains(Tigit)), whereas exported CD8^+.^T cells are enriched for a gene set that is downregulated upon exhaustion (e.g., transcription factor 7(Tcf7), selectin L(Sell), Lympho-enhancing factor 1(Lef1), chemokine receptors 7(Ccr7), and Sphingosine-1-phosphate receptor 1(S1pr1)) (92). In addition, CD8^+^ T cells within the tumor predominantly express TCF1. However, a subset of exported CD8^+^ T cells expresses both TCF1 and PD-1, with PD-1 exported cells expressing lower levels of PD-1 than retained cells (93, 94). Finally, a fraction of the exported CD8^+^ T cells could produce effector cytokines after restimulation *in vitro*, whereas the retained CD8^+^ T cells could not produce effector cytokines (95).

## Relationship between immunotherapy and tumor-associated lymphatic vessels

In the immunotherapy of Tumor-associated lymphatic vessels, we focused on the role of VEGF. VEGF and its receptor are mainly responsible for the occurrence, development, and remodeling of lymphatic vessels (96). The major molecular drivers of tumor-associated lymphatic vessels are VEGF-C and VEGF-D, which are produced by tumor and infiltrating myeloid cells (96). VEGF-C and VEGF-D exert their biological effects by binding to VEGFR-3 and VEGFR-2, and activate receptor tyrosine kinase activity through autophosphorylation, thereby activating the function of lymphatic endothelial cells (LECs) and tumor-associated lymphatic vessels (97, 98). The blockade of the VEGF-C/VEGFR-3 pathway inhibits tumor-associated lymphatic vessels and tumor growth in colorectal and breast cancer models (99). In both glioblastoma and intracranial melanoma models, VEGF-C delivery is highly synergistic with immune checkpoint inhibitor treatment (anti-PD-1 alone or in combination with anti-CTLA-4) (100). Many chemokines have been found to have different effects on tumor-associated lymphangiogenesis. For example, C-C motif chemokine ligand 5(CCL5) is involved in angiogenesis and can increase VEGF expression in tumor cells by activating chemokine receptors 1 and 5 (CCR1 and CCR5) (101–104). Moreover, it has a synergistic effect with C-C motif chemokine ligand 4(CCL4) to indirectly cause lymphangiogenesis by increasing the expression of VEGF-C (105–107). Clinical and histopathological studies showed that cyclooxygenase-2 (COX-2) expression was associated with tumor-associated lymphatic vessels density and lymph node metastasis in human malignancies (108–111). The treatment with COX-2 inhibitors can halt tumor progression by simultaneously inhibiting local inflammation, tumor-associated lymphatic vessels, and angiogenesis (112, 113). Also, evidence shows that IFN-γ secreted by cytotoxic T cells reduces lymphatic vessel density in homeostatic and inflamed lymph nodes(LN) and promotes the immunosuppressive function of LECs in tumors (80, 82, 114). Therefore, targeted therapy increases the number of tumor-associated cytotoxic T cells, thus inducing the apoptosis of LECs and decreasing the number of tumor-associated lymphatic vessels (81).

## Relationship between TA-HEVs and tumor-associated lymphatic vessels

HEVs are special vessels for transporting lymphocytes. They belong to the tumor-associated vasculature together with tumor-associated lymphatic vessels (115). Therefore, relevant treatment options may play a role in both HEVs and tumor-associated lymphatic vessels at the same time. For example, previous studies have shown that using anti-PD-L1 immunotherapy in combination with antiangiogenic therapies (anti-VEGF or anti-VEGF/Ang2) resulted in a mutually beneficial effect in targeted antiangiogenic immunotherapy. More importantly, antiangiogenic immunotherapy successfully induced the generation of HEVs and inhibited the generation of tumor-associated lymphatic vessels. Moreover, the vascular normalization induced by antiangiogenic therapy increased lymphocyte infiltration and activation (75, 116, 117). Therefore, ICB combined with antiangiogenic drugs can be used in immunotherapy to simultaneously induce HEV production and tumor-associated lymphatic vessels inhibition. Another association between HEVs and tumor-associated lymphatic vessels is that they are both involved in transporting lymphocytes in the tumor tissue. Therefore, treating lymphocytes, especially CD8^+^ T cells, may simultaneously affect this complex trafficking mechanism. However, the exact mechanism needs exploration.

## Summary and prospect

In this review, we focused on the entry and exit of lymphocytes into and out of tumor tissue through TA-HEVs and tumor-associated lymphatic vessels, respectively, and their impact on immunotherapeutic approaches. The special structure and function of TA-HEVs account for their role as the main site of lymphocyte extravasation into tumors during cancer immunity and immune checkpoint inhibitor immunotherapy. TA-HEVs have been identified in various human tumor tissues as a major portal for lymphocyte entry into the tumor and have been found to associate with TLS-rich regions. The induction of TA-HEVs may be associated with the recruitment of CD8^+^ T cells and may exert an inhibitory effect on tumor growth. However, tumor-associated lymphatic vessels play an important role in controlling the exit of lymphocytes from tumor tissues. The lymphatic vessel system controls the exit of lymphocytes from the tumor mainly through the synergistic differentiation of various chemokines and T cells, thus controlling the function and number of CD8^+^ T cells in the tumor immune microenvironment and inhibiting the immune effect to a certain extent.

Tumor cells evade human immune system surveillance by constructing various biophysical and biochemical barriers to block entry and promote the expulsion of immune cells such as lymphocytes. Also, lymphocytes are the key to tumor immunotherapy. The promotion of MECA-79^+^ TA-HEV production and the inhibition of CXCL12 work together to regulate the transport of lymphocytes in and out of the tumor as well as the number of immune cells such as CD8^+^ T cells and DCs, resulting in better therapeutic effect and prognosis for ICB combined therapy and other methods.

In summary, this review highlighted the importance of TA-HEVs and tumor-associated lymphatic vessels in immunotherapy and analyzed the two potential pathways regulating the flow of lymphocytes into and out of the TME. The modulation of these channels may be important for improving the efficacy of immunotherapy.
